# Report of the Hospital at Ningpo for 1852; under the Medical Missionary Society in China

**Published:** 1854-06

**Authors:** 


### BIBLIOGRAPHICAL NOTICES.

*Report of the Hospital at Ningpo for* 1852*; under the Medical Missionary Society in China.* By DANIEL J. MACGOWEN, M. D. Canton. 1852.

The author of this interesting report, is an American Missionary physician, stationed at Ningpo, China, under the patronage of the Baptist Missionary Union of the United States. While his ostensible office is to preach the Gospel to the nations of China, with the hope of remedying their spiritual condition, he is ardently devoted to the science of medicine, and in his zeal and benevolence to do good, the promptings of his nature have led him to devote a portion of his time to the relief of the bodily ailments of the people among whom he labors. These services have not only been highly appreciated but already extensively useful. Hitherto, his reports have been made through the proceedings of the society at Hong Kong, but hereafter, by a new arrangement, they will appear in a separate form.

The report regrets the limited means of the Society, which do not enable them to furnish provision for the treatment of in-door patients; all they do and all they contemplate at present, is through a Dispensary arrangement. In this way, however, they have rendered timely aid during the year to "seven thousand nine hundred and fifty six" persons, among "the sick poor of the city and environs." The principal diseases treated were "Opthalmia" and "Cutaneous affections," which are exceedingly prevalent in China; of the former there were 3856, of the latter 1989—in all, over 70 per cent of the whole number of cases under treatment.

In the autumn of 1848 Rubeola appeared epidemically in Ningpo, and throughout that province, but not in a malignant form; it also "prevailed in the maritime districts of the east coast of China and throughout the whole Pacific coast," and in some places was particularly fatal. In the Russian colonies, great numbers were carried off. In a periodical, "The Friend," printed at Honolulu in the Sandwich Islands, a Russian Captain reports thus: "some of our Islands in the Alsatian chain lost most of their population. In Sitka, amongst a population of 600, we had in one month eighty deaths, if not more." At the Sandwich islands it was also very destructive among the natives. In concluding this subject the report says, "it is remarkable, that whilst Rubeola was traversing this region of the earth, from the tropic of cancer to the frigid zone, the cholera was pursuing a western course, from the Volga to the Mississippi."

In 1852, like many other countries, this province was visited by cholera. As the report presents a very interesting account of this epidemic, as well the novel theory of its pathology and mode of treating it, as taught by the Chinese doctors, we prefer to give it in the language of Dr. Macgowen himself:

"Finally that modern scourge, 'whose symptoms begin with death,' cholera asphyxia, has been in our midst. In its eastern march this disease reached China through the Straits in 1820. During the summer of that and the following year, Ningpo, like other portions of the empire, suffered severely. Since the last named period, it has not prevailed epidemically, though few years pass by without the occurrence of sporadic cases. On its reappearance this autumn, it was instantly recognized as the *kioh-kin-tiáu* or the disease which 'contracts the tendons of the leg,' the name given to cholera soon after it was first observed. Its appearance occasioned but little alarm or excitement. A short time before its disappearance, the gods were carried in procession through the streets, and propitiatory offerings made in several places.

There was one gratifying circumstance connected with the prevalence of the cholera, which I here mention with much pleasure. Great pains were taken by benevolent persons to make public those remedies which were considered best adapted to arrest the disease in persons attacked. Placards were posted in every quarter, giving directions for the treatment of the malady in its different phases. All recommended substantially the same mode of treatment, which seems to have been taken from a small monograph on cholera by a physician of Kiáhing, Sü Tsz'mí. Dr. Sü states that on the first appearance of the disease, medical men took it for ordinary cholera, and treating it accordingly, failed to save one in a hundred of their patients; but observing that the disease arose from derangement of the three *ying* (stomach, lungs, and kidney,) he reversed the practice, and employed remedies for warming or stimulating the vessels. He regarded the disease to arise from 'morbific cold,' disturbing the harmony naturally subsisting between the dual powers of the system. His professional brethren contended that 'accumulated heat' destroyed the equilibrium existing between these two powers, and whilst he relied on stimulants, the others resorted to cooling remedies. Our author's system of course prevailed, for though it often failed to cure, it never killed the patient, which the rival system could not fail to do.

To impart vital energy and warmth to the body, the juice of fresh ginger was given, and to this pungent stimulant, various aromatics and bitters were added. Ginger and ginseng entered into every formula employed, but it was generally stated that the latter being expensive, might be dispensed with. As a preliminary step, sternutatories were employed, and if the patient could be made to sneeze, he was considered to be in a more favorable condition than if insensible to such stimulants. Counter-irritants also were resorted to, composed of salt and garlic, which, with moxa, were applied over the abdomen, and for the same purpose foot-stoves were used for the extremities. The feet and legs also were rubbed and shampooed. Thus, despite their fanciful theories, Chinese physicians pursued the same therapeutic course, which in the West have been found most efficacious. Some attached considerable importance to acupuncture, in dangerous cases piercing the tips of the tongue, fingers, and toes, but particularly the popliteal space. It was stated that the tendon in that place would present a livid appearance for the space of an inch, if it were a case of pure cholera. A silver needle was to be thrust to the depth of the eighth of an inch, and left in during the space of time occupied in six inspirations, and the dark blood which would flow from the incision would tend to restore the equilibrium of the dual powers.

By relying on such means, native practitioners afforded relief to many patients, but they were powerless when attempting to treat the consecutive fever, and hence the mortality was very great. The cases which came under my observation presented all the marks of Indian cholera in a most striking manner; vomiting and purging of a congee-like fluid, painful cramps, low hoarse voice, livid skin, rapid prostration, sunken eyes, restlessness, a shriveled appearance of the whole body, cold perspirations, and finally collapse. How far the disease has extended this season in China, it is impossible at present to ascertain. It prevailed at Hángchau several weeks before it reached the prefecture of Ningpo. Somewhat later it is said to have been seen in the neighborhood of Shánghái. It is remarkable that the villages of the plain of Ningpo suffered most, affording another evidence that in this part of China, at least, the cities are the most healthy portions of the land. The fevers so common here, whether intermittent, remittent, or typhus, are far more frequently met with in the rural districts, than in the cities. As regards my patients suffering from these diseases, above eighty per cent. have been countrymen and villagers, much of this may be owing, perhaps, to the greater destitution of the suburban population, which impels them to apply to me for relief, but it is mainly owing to the less salubrious state of the country.

The filthy condition of the city, its stagnant canals, exposure of nameless ordures, the number of dead left in coffins above ground in vacant spaces, would seem to render this like other Chinese towns the very focus of malaria; but while this state of things is unfavorable to longevity, it has not, so far as observation extends, caused them to be peculiarly obnoxious to epidemics. In the cities, drainage is more perfect than in the country; and citizens possess a larger share of domestic comfort and better means of subsistence than the inhabitants of secluded places. Generally speaking, the most salubrious sites are those immediately adjacent to the cities, and yet sufficiently removed from fields and gardens."

Small Pox, it appears, has not been unfrequent at Ningpo. Little attention having been given to vaccination, while at Canton, it has been largely practiced.

The most animating portion of this report, however, is that which refers to the treatment and cure of those Chinese who have been addicted to the fascinating yet destructive vice of smoking opium; many hundreds of whom the report tells us, are living witnesses of his success, in the means employed for their relief. Not a few of these, the doctor says, "are of several years standing; men too, who are occupied all day in labor, requiring great muscular activity, and from whom every vestige of the characteristic features of the opium victim have been effaced. Some whose vital powers were nearly exhausted, are now stalwart chair-bearers, and once more in the enjoyment of existence."

It is, however, only among the more resolute, and those who have become outcasts from society, and beggared by the impoverishing vice, that the rigid measures adopted have been triumphant. No anodyne is substituted by Dr. Macgowen for the deprivationof the fascinating stimulus, except Dover's powders —which is only prescribed to check the wasting diarrhœa which invariably follows abstinence from opium in the habitual smoker. Native practitioners attempt to cure by opiates in graduated doses, but their subjects are liable to relapses, when, it is said their condition is more hopeless than ever.

This part of the report would have been rendered far more interesting and profitable to the medical reader, if Dr. Macgowen had given the particulars of his manner of treating these opium victims. He speaks of "the stringent conditions with which they are made to comply," of the agony they endure, and that during this period "they are sustained by various stimulants," but what these stimulants are, or the stringent conditions required of them in order to their reception as patients or the length of time required to effect a cure, we are left to conjecture.

It is to be hoped, however, that the intelligent author of this report, will hereafter give to the profession a succinct account of his method of treating a disease, national in its character and threatening to ruin both state and people. Alluding to the tardiness with which the science of surgery advances, the report goes on to say:

"In operative surgery with the exception of the ophthalmic department, little has been accomplished. Cases requiring capital operations are rarely met with, and there exists, moreover, a greater repugnance to the knife in this part of China, than at Canton. Tumors are comparatively uncommon. The greater part of operations on the eyes have been for entropion, which is remarkably prevalent."

Gunpowder injuries are spoken of, as of frequent occurrence, the result of carelessness and awkardness, in their pyrotechnic amusements and in their military exercises. One interesting case is named where a poor fellow, already a cripple, dragged himself near to a perpendicular iron tube containing a rocket which had failed to go off. Looking into the tube the rocket exploded, destroying both his eyes, carrying away part of his forehead and destroying a portion of the brain. From this severe injury the man recovered without having his mental faculties impaired.

In conclusion, the report states that "a prominent object of professional pursuit here has been the instruction of native practitioners and students in anatomy and the sciences of the healing art. Heretofore this has been done to a limited extent only, owing to the want of a lecture room. The rooms of Moon Lake College have been placed at my service, and have been occasionally employed for delivering astronomical lectures; but even for this purpose it is ill adapted. It was impossible to exercise any control over the admission of spectators."

We are glad of an opportunity to learn even a little of the comparatively unknown system of medicine in China. The subjects treated of in this report deserve a more extended investigation, and we hope that the author, who is a man of science, as opportunities are afforded him, may have the disposition to complete the work he has with much earnestness commenced, and not only make "the benefits of the healing art more extensive and effective than heretofore" in China, but add something to the medical literature of the day by his physiological and pathological researches.

